# Thin films of formamidinium lead iodide (FAPI) deposited using aerosol assisted chemical vapour deposition (AACVD)

**DOI:** 10.1038/s41598-020-79291-1

**Published:** 2020-12-17

**Authors:** Firoz Alam, David J. Lewis

**Affiliations:** 1 Department of Chemistry, The University of Manchester, Oxford Road, Manchester, M13 9PL UK; 2 Department of Materials, The University of Manchester, Oxford Road, Manchester, M13 9PL UK

**Keywords:** Energy science and technology, Materials science

## Abstract

Formamidinium lead iodide (CH(NH_2_)_2_PbI_3_, FAPI) thin films have been deposited on glass substrates at 150 °C using ambient pressure aerosol assisted chemical vapour deposition (AACVD). The films have been analysed by a range of techniques including powder X-ray diffraction (pXRD), scanning electron microscopy (SEM), energy dispersive X-ray (EDX) spectroscopy, and UV–Vis–NIR absorption spectroscopy. Sharp reflections in the pXRD pattern can be indexed to the α-phase of FAPI which confirms the crystallinity of the as-deposited film and reveals a preferred growth orientation along the (002) plane with respect to the substrate. High magnification SEM images show that the thin film is comprised of a network of intimately connected FAPI crystallites which form a mesoporous architecture. EDX mapping of lead and iodine emission peaks show that the Pb and I within these films are spatially co-localised. Optical measurements show as-deposited FAPI films have absorption onsets in the near infra-red with a direct bandgap value of 1.46 eV, suitable for single junction solar cells. Four-point probe measurement of as deposited films show that the electrical conductivity (σ) of the FAPI thin film is 5.2 × 10^–7^ S/cm, which is similar to FAPI thin films deposited by spin coating technique.

## Introduction

Metal–organic lead halide perovskites with general formula ABX_3_, where A = methylammonium (CH_3_NH_3_^+^ or MA), formamidinium (CH(NH_2_)_2_^+^ or FA); B = lead (Pb^2+^); and X = chlorine (Cl^−^), bromine (Br^−^), or iodine (I^−^) have emerged as a very promising class of materials for thin film photovoltaics^1–4^. Solar cells utilising mixed halide Pb-containing perovskites as a light absorber layer have achieved a certified power conversion efficiencies of over 25% for single junction devices^5^, and significant progress has been made to integrate perovskite thin films into tandem solar cells architecture with silicon. A certified power conversion efficiency of 28% for a 1 cm^2^ area perovskite-on-silicon tandem solar cell has recently made the prospect of power conversion efficiencies greater than 30% using multijunction solar cell technology a real possibility^6,7^.

Whilst these perovskite solar cells based on methylammonium lead iodide (CH_3_NH_3_PbI_3_ or ‘MAPI’) have shown excellent efficiencies, they also suffer from instability particularly in the presence of water, where deprotonation of the ammonium group leads to the formation of HI. Formamidinium lead iodide (CH(NH_2_)_2_PbI_3_ or ‘FAPI’), however, has attracted great attention because of its higher thermal, moisture, and chemical stability, from the relatively inert formamidinium proton^8^. In addition, replacement of the methylammonium (CH_3_NH_3_^+^) cation in MAPI with a slightly larger formamidinium (CH(NH_2_)_2_^+^) cation in FAPI decreases the optical bandgap from 1.54 to 1.47 eV, due to changes in cation radius, and extends the absorption into the near-infrared^9,10^. Hence, power conversion efficiencies in excess of 20% have been reported with FAPI with the benefit of the materials being far more stable under ambient conditions^11^.

It is reported in the literature that FAPI crystallises into two polymorphs: (i) the black cubic perovskite phase (α-phase) with space group Pm-3 m; and (ii) the yellow non-perovskite hexagonal phase (δ-phase) with space group P6_3_mc^9,12^. Polycrystalline thin films, as well as single crystals of FAPI, slowly transform to the non-perovskite hexagonal yellow δ-phase at room temperature. Heating the material above the phase transformation temperature (*T* > 150 °C), promotes a reverse transformation from the yellow to the black phase^8^. The polycrystalline black materials of the α-phase obtained from heating a crystal of the δ-phase do not convert back to the yellow phase on cooling over any appreciable time period. It has been suggested that this is  due to the partial loss of formamidinium ions resulting in cation defective materials^13^.

Several methods have been used to deposit hybrid perovskite thin films^14–16^, yet the preparation of large area perovskite films using a cost-effective technique is still a significant challenge. Spin coating is widely used for perovskite solar cells fabrication because of its simplicity, but uniformity over large areas is difficult to achieve^14^. A two-step solution based route has also been used to deposit perovskite films, which includes deposition of PbI_2_ using spin coating followed by heating of the PbI_2_ film at 70 °C for 30 min. The film was then immersed into a solution containing formamidinium iodide (FAI) for 15 min, and was rinsed with isopropanol (IPA) and spun at 4000 rpm for 30 s^16^. Vapour assisted solution processing (VASP) has also been used to obtain thin films of hybrid perovskites, in which a PbI_2_ film was deposited by spin coating technique and dried at 110 °C for 15 min. Methylammonium iodide (MAI) powder was spread out around PbI_2_ film with a petri dish covering on the top, and heated at 150 °C for desired time. After cooling down, the film was washed with IPA and then dried and annealed^17^. The so-called Direct contact intercalation process (DCIP) has also been used to obtain perovskite films, where a PbI_2_ film was deposited by spin coating followed by annealing at 70 °C for 15 min. A flat covering of methylammonium iodide (MAI) powder was spread onto the bottom of an aluminium container preheated at 150 °C, onto which PbI_2_ film was directly pressed. The intercalation reaction was performed for specific time durations at different temperatures to effect the conversion of the PbI_2_ film to a MAPbI_3_ film. After cooling to room temperature, the final film was washed with IPA and dried under a stream of nitrogen^18^. However, all of these alternatives are often problematic due to poor stoichiometry as unreacted PbI_2_ remains in the final films and they are challenging to scale-up. Jiang et al. reported a perovskite film deposition method combining raster ultrasonic spray (RUS) coating and chemical vapour deposition (CVD), where uniform PbI_2_ films were prepared by RUS using a precursor solution containing a solvent mixture of DMF and DMSO. PbI_2_ films were then converted to the corresponding hybrid perovskites by CVD in the presence of FAI, FABr and MACl vapour^19^. Vacuum based vapour deposition techniques are viable for multilayer large area, highly uniform, pinhole-free, smooth thin films as it does not require solvent, but the requirement of high vacuum systems makes them prohibitively expensive in many cases^20^. A table showing the advantage of the AACVD method over other reported methods for producing perovskite films has been incorporated into the Supporting Information of this paper.

Combining the benefits of solution and vapour based methods mentioned above, aerosol-assisted chemical vapour deposition (AACVD) has been used extensively for the production of transparent conducting oxides (TCOs)^21^, layered materials^22^, metal chalcogenides^23,24^ as well as perovskite thin films^25–27^ on a range of substrates. In 2014, Lewis et al. first reported the deposition of phase pure CH_3_NH_3_PbBr_3_ perovskite thin film on glass substrates at 250 °C using single step AACVD technique^25^. Later on, Palgrave et al. reported deposition of CH_3_NH_3_PbI_3_ perovskite thin film on glass substrates at 200 °C using AACVD^27^. Binions et al. reported a two-step sequential AACVD technique which deposited extremely pure, uniform and pinhole-free thin films of CH_3_NH_3_PbI_3_ on fluorine-doped tin oxide (FTO) glass substrates at 220 °C^28^. Revaprasadu et al. reported the deposition of CsPbBr_2_I perovskite films on glass substrates at 100 °C using AACVD from the precursor solution containing olelyamine as a capping agent^29^. Flavell et al. reported the deposition of Cs_2_SnI_6_ and MAPI perovskite films on ITO and glass substrates at 130 °C using AACVD and spin coating and studied their stability using near ambient pressure XPS, giving insight into the degradation mechanism in the presence of water^26,30^. In this communication, we report for the first time the deposition of thin films of FAPI on glass substrates using AACVD from a single precursor solution. Importantly, we show that such films have a band gap commensurate with solar light harvesting and are comparable to films produced by spin coating in terms of their electrical characteristics. AACVD is an ambient pressure CVD technique that is simple, cost-effective, proceeds in a single step and has previously been adapted by industry for assembly-line glass coating (e.g. Pilkington). Hence, we demonstrate proof of principle for this first step towards fabrication of large area photovoltaic devices.

## Results and discussion

**Figure 1:**
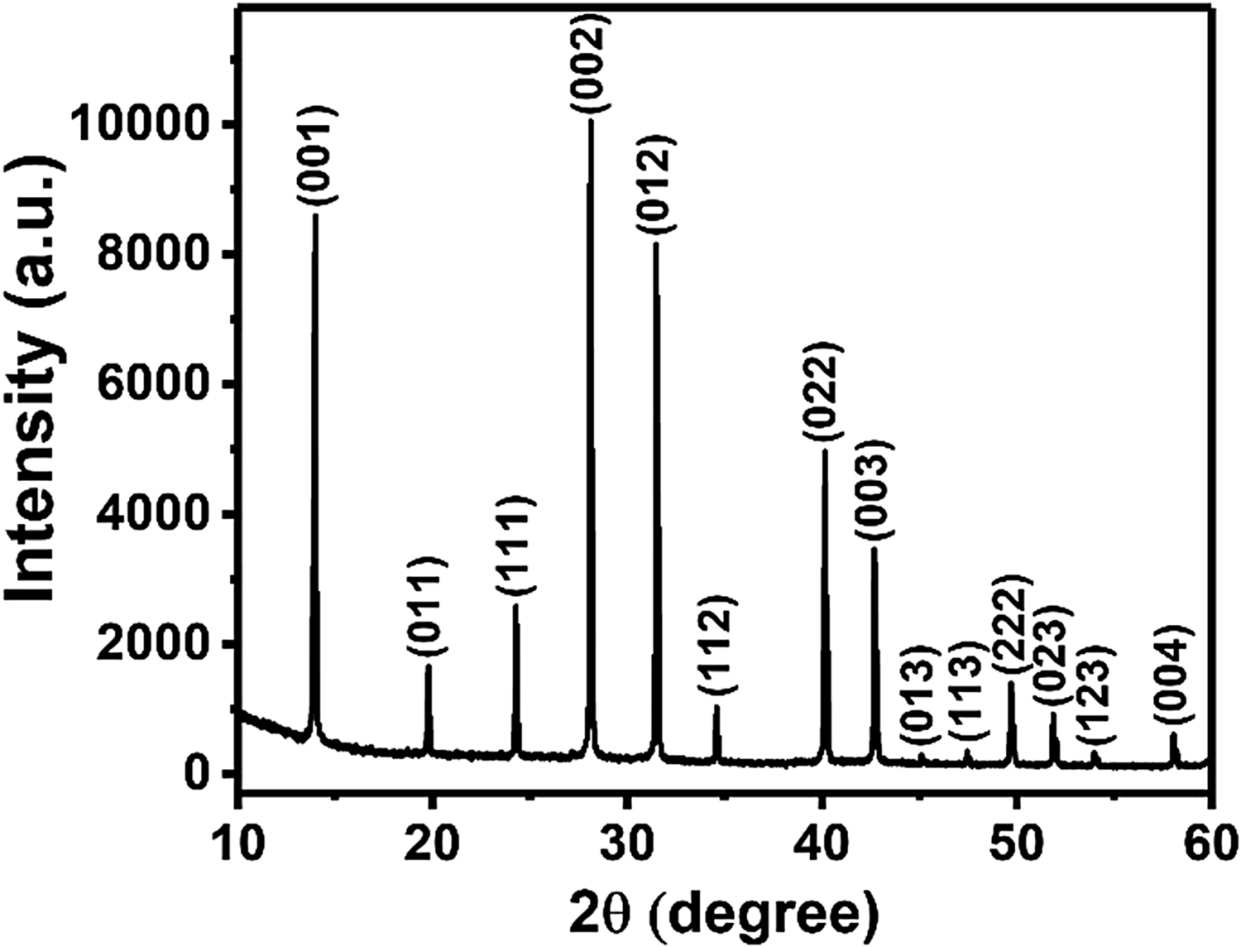
Powder XRD pattern of a FAPI thin film deposited by AACVD on a glass substrate at 150 °C.

Solutions for AACVD were obtained by dissolving lead iodide (PbI_2_) in *N*,*N*-dimethyl formamide (DMF) with stirring at 70 °C for 1 h and then the as-synthesised formamidinium iodide was added to the solution with continuous stirring for another 20 min at same temperature. The precursor solution was then filtered using a PTFE membrane (0.45 µm, Ossila) prior to use. An aerosol mist of the precursor solution was generated using an ultrasonic humidifier, which was then transported using a stream of argon gas (250 sccm) into a hot wall reactor at 150 °C containing glass substrates. After decomposition of the precursor a dark black film of FAPI was obtained. The as-deposited thin film on glass substrate was characterised using powder X-ray diffraction (pXRD) in the range of 10° < 2θ < 60° (Fig. 1). Reflections corresponding to the (001), (011), (111), (002), (012), (112), (022), (003), (013), (113), (222), (023), (123) and (004) planes of cubic CH(NH_2_)_2_PbI_3_ perovskite (α-phase) were observed and are consistent with those previously reported in the literature^9,12^. The enhanced (002) peak with respect to (001) and (012) peaks indicate a preferred crystallographic orientation in the film with respect to the substrate. It is also observed that after storage for two weeks in a glovebox, we do not observe any change in the films visual appearance which means negligible conversion from the black to the yellow phase took place. To confirm this, the pXRD pattern of the sample after the same time period is unchanged and does not show any new peaks of PbI_2_ or other impurity peaks (Fig. S1).

**Figure 2:**
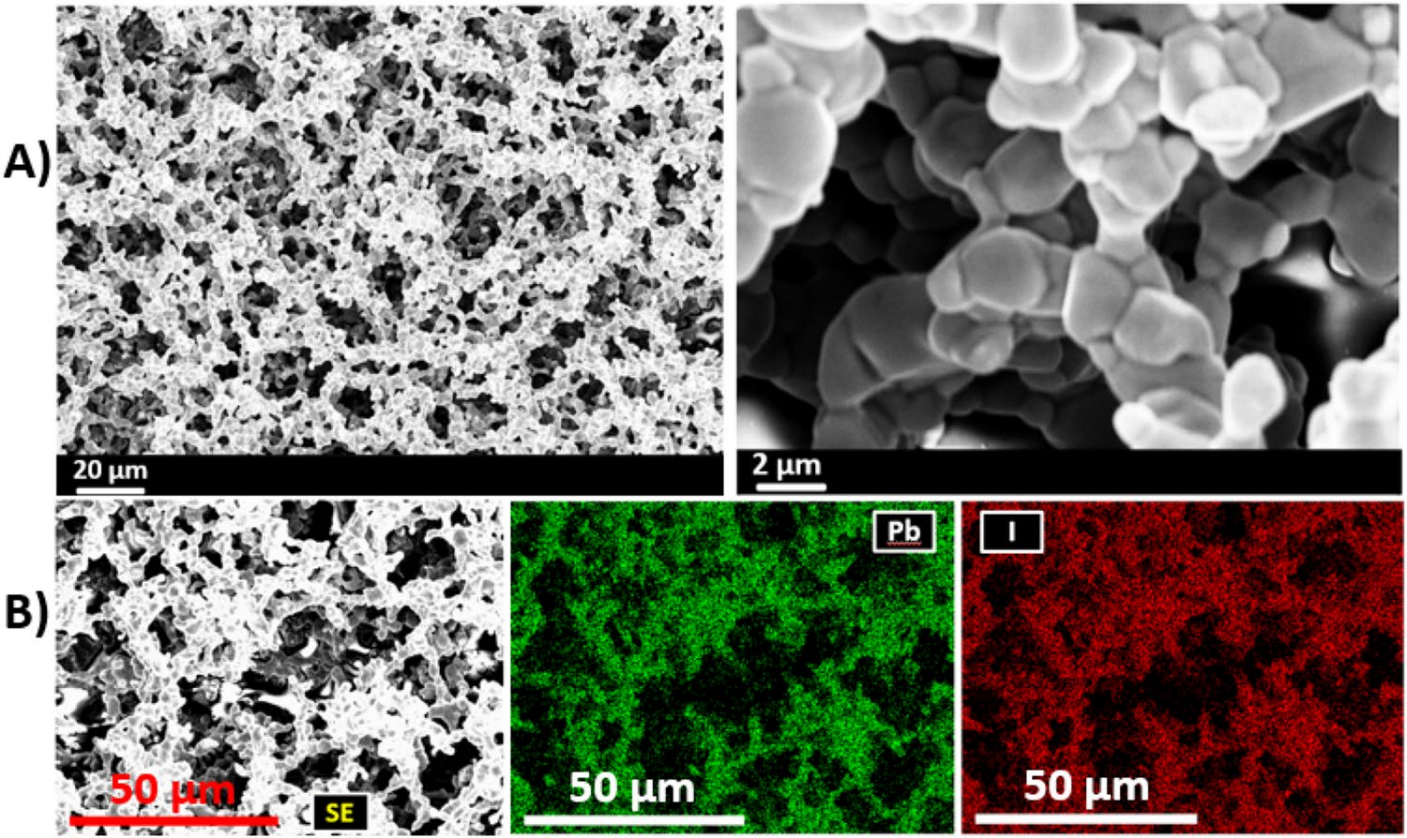
Investigation of surface morphology of FAPI thin films and spatial elemental distribution of Pb and I within the films. (**A**) Secondary electron SEM images taken at low and high magnifications showing gross mesoporous appearance and interconnectivity of FAPI crystallites. (**B**) EDX spectroscopic mapping of FAPI showing the distribution of the Pb and I (mapped from K alpha emission intensity) within the film in plan view. The elements are co-localised in space.

The surface morphology of as-deposited thin films of FAPI was investigated using scanning electron microscopy (SEM) in secondary electron mode at an acceleration voltage of 10 keV (Fig. 2A). Low magnification SEM images show a well-connected network of crystallites with a high surface area mesoporous architecture, whilst higher magnifications reveal that these networks are comprised of interconnected large crystalline grains of FAPI with domain sizes in the range of 1–2 µm. The interconnected nature of the crystallites within the thin film perhaps leads to the acceptable electrical characteristics of these films that we observe via four point probe measurements (vide infra). Elemental mapping using energy dispersive X-ray (EDX) spectroscopy at an acceleration voltage of 10 keV showed that the distribution of lead (Pb) and Iodine (I) is spatially co-localised over the interrogated area of ca. 100 × 75 µm (Fig. 2B).

**Figure 3:**
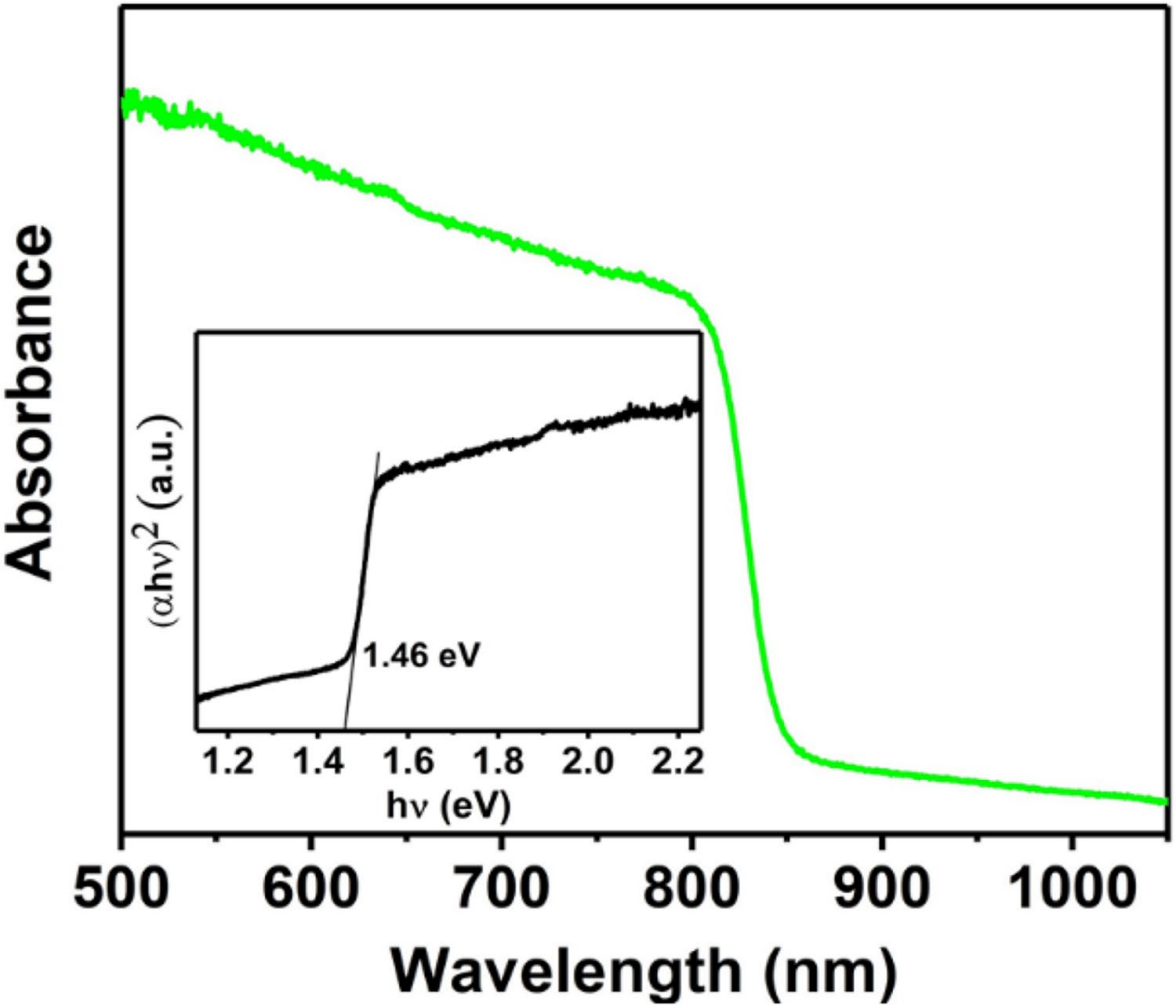
Optical absorbance spectrum of a FAPI thin film deposited by AACVD on a glass substrate at 150 °C. Inset: Tauc plot of FAPI thin film showing an estimated direct optical bandgap of 1.46 eV.

The UV–Vis–NIR absorption spectrum of as-deposited FAPI on glass substrates in the wavelength range of 500–1050 nm is shown in Fig. 3. Strong optical absorption is observed in the wavelength range of 500–860 nm. An estimation of the optical bandgap of FAPI films, was made using a plot of (αhν)^2^ vs hν (Tauc method), by extrapolation of the linear region of the absorption edge to the energy axis, with the intercept indicating a direct bandgap of 1.46 eV, which is in a good agreement with the previously reported literature values^10,31^. It has been reported that replacing methylammonium in MAPI with the slightly larger formamidinium ion in MAPI narrows the bandgap values closer to the optimum bandgap for single junction solar cells, and our results here mirror this trend^31^.

In order to demonstrate their viability as charge carriers, we studied the electrical properties of the AACVD deposited FAPI thin films on glass substrates using a four-point probe measurement. The electrical properties of FAPI films are shown in Table 1. The obtained electrical conductivity (σ) is 5.2 × 10^–7^ S/cm, which is in good agreement with the previous report by Yang Yang and co-workers^32^. We believe that the excellent conductivity of these films is due to the interconnected nature of the FAPI crystallites within the mesoporous film that we observe by high magnification SEM (vide supra).

**Table 1:** Electrical properties of CH(NH_2_)_2_PbI_3_ nanostructures deposited by AACVD on glass substrate at 150 °C.

Sample	Sheet resistance (GΩ/□)	Film thickness (μm)	Electrical conductivity (S/cm)
CH(NH_2_)_2_PbI_3_	3.98	4.8	5.2 × 10^–7^

## Conclusions

In summary, AACVD has been used for the deposition of hybrid perovskite thin films on glass substrates. Sharp reflections in the powder XRD pattern can be indexed to FAPI and confirm the crystallinity of the as-deposited hybrid perovskite with a preferred orientation along the (002) plane with respect to the substrate. High magnification secondary electron SEM images show that the FAPI crystallites produced are large and in the range of 1–2 µm diameter and form a network of intimately connected grains that form a mesoporous architecture. EDX spectroscopic mapping of lead (Pb) and Iodine (I) with secondary electron SEM image of the same area showing that the Pb and I within these films are co-localised at the nanoscale. UV–Vis–NIR absorption spectroscopy of as deposited FAPI on glass substrates show strong optical absorption in the wavelength range of 500–860 nm with estimated direct bandgap value of 1.46 eV, suitable for single junction solar cells, and the electrical properties are similar to FAPI films produced by spin coating, and hence we are confident that materials produced by this method could be viable for coating large areas with photoactive FAPI. We also note that the AACVD technique does not require post annealing or high vacuum and proceeds in single step and thus has great potential for scale-up.

## Methods

Formamidinium acetate powder and hydroiodic acid (HI) were purchased from Merck. Lead iodide (PbI_2_), N,N-dimethyl formamide (DMF), ethanol and diethyl either were purchased from Sigma-Aldrich. All the chemicals were used as received without any further purification.

### Synthesis—formamidinium iodide (FAI)

Formamidinium iodide (FAI) was synthesised by dissolving formamidinium acetate powder (10.41 g) in 19.72 mL of 57% w/w hydroiodic acid (HI) with stirring at 50 °C for 30 min. The solution was then stripped to dryness at 70 °C for 2–3 h in rotary evaporator, leading to dark brown crystalline solids. Upon drying at 100 °C for 1 h, a yellow-white powder is formed. This was then washed with five times with diethyl either. The powder was then fully dissolved in ethanol with stirring and heated at 80 °C for 10 min. After that the solution was placed in a refrigerator for overnight for recrystallization. This recrystallization process forms white needle-like crystals. These white needle-like crystals were washed with diethyl either three times and were then dried overnight in a vacuum oven at 60 °C. Elemental analysis for FAI (%): Calc. (Found) C, 6.98 (7.12); H, 2.93 (2.86); N, 16.29 (16.14); I, 73.80 (73.65).

### Precursor solution

PbI_2_ (2.02 g, 0.88 M) was dissolved in 5 mL of N,N-dimethyl formamide (DMF) with stirring at 70 °C for 1 h and then FAI (0.756 g, 0.88 M) was added to the solution with continuous stirring for another 20 min at same temperature. The precursor solution was then filtered using PTFE (0.45 µm, Ossila) filter before use.

### Aerosol-assisted chemical vapour deposition of CH(NH_2_)_2_PbI_3_ thin films

FAPI thin films were deposited on cleaned glass substrates (1.5 cm × 3 cm) using aerosol-assisted chemical vapour deposition technique from a clear solution mentioned above. The apparatus for preparation of perovskite film in the laboratory is shown in Fig. S2 and has been previously described by Ramasamy et al.^23^ The flow rate of the argon carrier gas was ~ 250 sccm and the tube furnace temperature was set to 150 °C. The AACVD run for 2 h upon which time the substrates were coated with dark black films of FAPI. The FAPI thin films were taken out from the tube furnace for further analysis, once the temperature is reached below 100 °C.

### Characterisation

Elemental analysis (EA) was performed using a Thermo Scientific Flash 2000 Organic Elemental Analyser by the University of Manchester micro-analytical service.

Powder X-ray diffraction (p-XRD) pattern of thin film deposited on glass substrate was measured using a Bruker AXS D8 Discover diffractometer, using copper Kα radiation (λ = 1.54178 Å). Films were scanned over the range of 2θ = 10°–60° using a step size of 0.02° with a dwell time of 0.5 s.

Scanning electron microscopy (SEM) imaging was performed using a Zeiss Ultra 55 FEG-SEM + EBSD + EDX in secondary electron mode with an accelerating voltage of 10 kV. Energy-dispersive X-ray (EDX) analysis was carried out using the same instrument with an accelerating voltage of 10 kV using an Oxford Instruments INCA pentaFETx3 detector. The thin films for SEM and EDX analysis were mounted on stubs using copper tape and earthed with silver paint.

UV–Vis–NIR absorption spectrum of thin film was recorded using a Shimadzu UV-1800 instrument over a range of 500–1050 nm.

Four-point probe measurement was performed to obtain the electrical conductivity of the film and Dektak-XT surface profiler was used for thickness measurement.

## Supplementary Information

Supplementary Information.
