# Supplementary material for: Thin films of formamidinium lead iodide (FAPI) deposited using aerosol assisted chemical vapour deposition (AACVD)
Source: Sci Rep. 2020 Dec 17;10:22245. doi: 10.1038/s41598-020-79291-1 (PMC7747716; doi:10.1038/s41598-020-79291-1)
Supplement: Supplementary file 1 — Supplementary Information. [file 41598_2020_79291_MOESM1_ESM.docx]

Electronic Supporting Information

**Thin Films of Formamidinium Lead Iodide (FAPI) Deposited using Aerosol Assisted Chemical Vapour Deposition (AACVD)**

Firoz Alam^1^ and David J. Lewis ^2^

^1^Department of Chemistry, The University of Manchester, Oxford Road, Manchester, M13 9PL, United Kingdom.

^2^Department of Materials, The University of Manchester, Oxford Road, Manchester, M13 9PL, United Kingdom.

Correspondence and requests for materials should be addressed to D.J.L (email: [david.lewis-4@manchester.ac.uk](mailto:david.lewis-4@manchester.ac.uk))


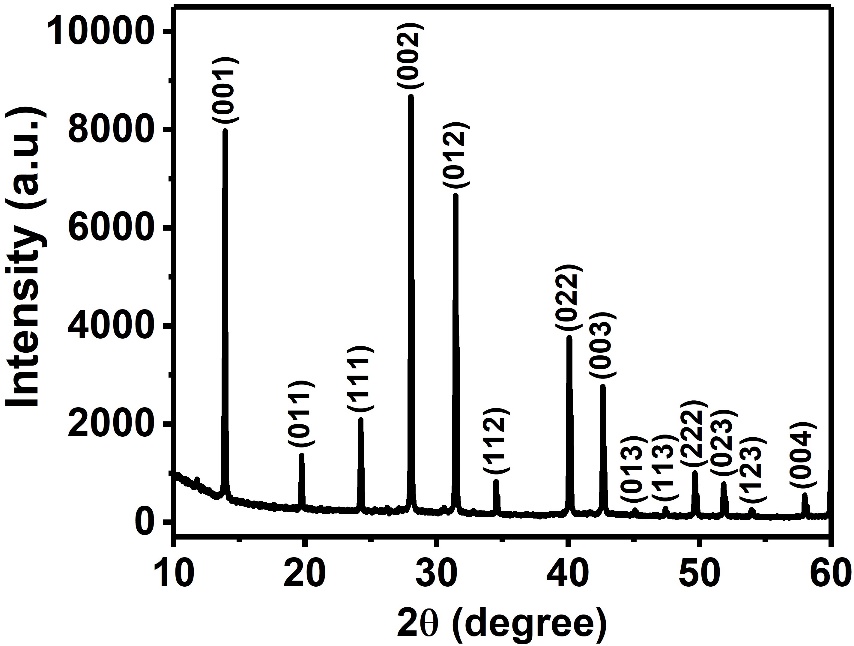


**Fig. S1** Powder X-ray diffraction pattern of as deposited FAPI film stored in a glovebox for two weeks.

**
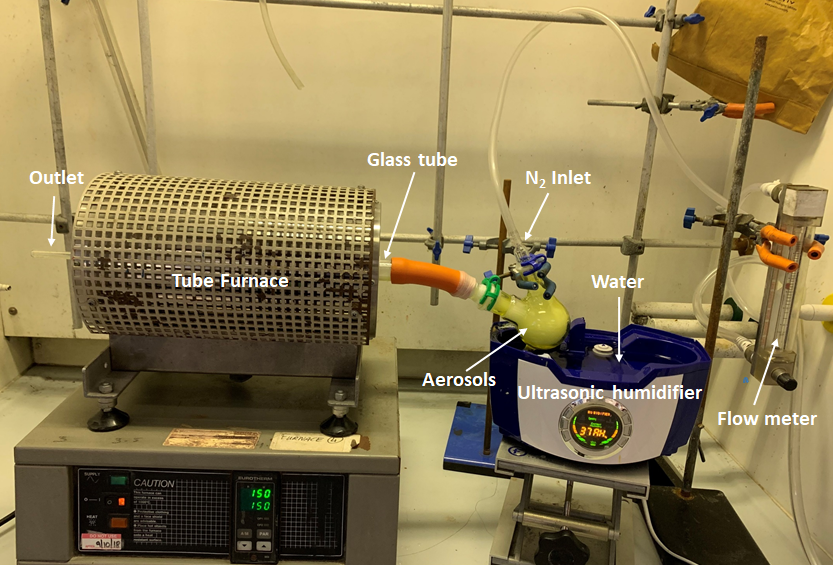
**

**Fig. S2** Picture of AACVD apparatus for preparation of perovskite film in the laboratory.

Table S1 shows the advantage of the AACVD method over other reported methods for producing perovskite film.

| Methods | Advantages | Disadvantages | Ref. |
| --- | --- | --- | --- |
| Aerosol assisted chemical vapour deposition (AACVD) | Work on ambient pressure, simple, cost-effective, eight substrates of dimensions ca. 3 cm × 1 cm laid out end-to-end can be deposited at a single time, proceeds in a single step and has been adapted by industry for assembly-line glass coating (e.g. Pilkington) and is suitable for the production of large area thin films on a range of substrates. |  | [1] |
| Spin coating | Widely used due to its simplicity, quickly and easily produce small area films, low cost, ease of set up. | Impossible to scale up because it is extremely difficult to obtain uniform  spin coated films over a large substrate, only allows one substrate at a time, reproducibility is very less and its two step technique, wastage of materials. | [2] |
| Chemical vapour deposition | High growth rate and reproducibility, good quality uniform films over large area, batch processing for higher throughput, low fabrication cost. | Requirement of high vacuum systems makes them less attractive, High capital cost. | [3] |
| Atomic layer deposition | High quality films, conformal growth of ﬁlms over large areas with atomic thickness precision, uniformity, low-temperature processing, stoichiometric control, multilayer, excellent repeatability. | Economic viability, high material waste rate, high energy waste rate, nanoparticle emissions, deposition rate slower than CVD. | [3, 4, 5] |
| Dual-source thermal evaporation system and sequential vapour deposition | Highly uniform,  pinhole-free, smooth thin films, does not require solvents, could overcome the sample size limit. | These techniques demands high  vacuum, which is too energy consuming and hinders mass  production. | [6, 7] |

**References**

1. Bhachu, D. S. et al. Scalable route to CH_3_NH_3_PbI_3_ perovskite thin films by aerosol assisted chemical vapour deposition. *J. Mater. Chem. A* **3**, 9071-9073, https://doi.org/10.1039/C4TA05522E (2015).
2. Eperon, G. E., Stranks, S. D., Menelaou, C., Johnston, M. B., Herz, L. M. & Snaith, H. J. Formamidinium lead trihalide: a broadly tunable perovskite for efficient planar heterojunction solar cells. *Energy Environ. Sci.* **7**, 982-988, https://doi.org/10.1039/C3EE43822H (2014).
3. Raiford, J. A. et al. Applications of atomic layer deposition and chemical vapor deposition for perovskite solar cells. *Energy Environ. Sci.* **13**, 1997-2023, https://doi.org/10.1039/D0EE00385A (2020).
4. Sutherland, B. R. et al. perovskite thin films via atomic layer deposition. *Adv. Mater.* **27**, 53-58, https://doi: 10.1002/adma.201403965 (2015).
5. Seo, S. et al. Atomic layer deposition for efficient and stable perovskite solar cells. *Chem. Commun.* **55**, 2403-2416, https://doi.org/10.1039/C8CC09578G (2019).
6. Borchert, J. et al. Large-area, highly uniform evaporated formamidinium lead triiodide thin films for solar cells. *ACS Energy Lett.* **2**, 2799-2804, <https://doi.org/10.1021/acsenergylett.7b00967> (2017).
7. Liu, M. Et al. Efficient planar heterojunction perovskite solar cells by vapour deposition. *Nature* **501**, 395-398, https://doi:10.1038/nature12509 (2013).
